# The Use of ChatGPT to Write Assessment Questions

**DOI:** 10.3390/pharmacy14070142

**Published:** 2026-09-24

**Authors:** Eytan A. Klausner, Karen S. Mark

**Affiliations:** Department of Pharmaceutical Sciences, South College School of Pharmacy, Knoxville, TN 37922, USA

**Keywords:** artificial intelligence, ChatGPT, course assessment, student academic performance, pharmacy education, pharmaceutics, Bloom’s taxonomy

## Abstract

It has been suggested that ChatGPT can be used to write assessment questions. This study aimed to evaluate student pharmacists’ academic performance on ChatGPT-generated questions in summative assessments, explore their perceptions regarding the use of ChatGPT to write assessment questions, and characterize the questions’ Bloom’s taxonomy levels. ChatGPT was used to generate assessment questions aligned with the lecture objectives. After selection by a content expert, ChatGPT-generated questions were used for all examinations in the Pharmaceutics I and Pharmaceutics II courses administered in 2024 and 2025. A survey administered to student pharmacists investigated their perceptions of using artificial intelligence (AI) to generate assessment questions. Ninety-one students participated in the study (response rate 93%). Students were graded on approximately 100 ChatGPT-generated questions for the combined courses. In both 2024 and 2025, in the Pharmaceutics I course, the average percent correct was higher on the ChatGPT-generated questions compared to instructor-generated questions, whereas in the Pharmaceutics II course, the percent correct was lower on ChatGPT-generated questions. Most students thought that instructors should not use ChatGPT to generate most (≥50%) or all their assessment questions. The average success rate of students in distinguishing between 20 assessment questions that were evenly split between ChatGPT- and instructor-generated questions was 53%. CompetencyGenie™, an AI-powered tool, assigned mostly Bloom’s taxonomy levels of Remember and Understand to the ChatGPT-generated questions. Student performance was similar across ChatGPT-generated questions from the two levels. ChatGPT can generate questions that are usable in summative assessments in pharmaceutics courses.

## 1. Introduction

Rapid developments in generative artificial intelligence (AI) are increasingly affecting a growing number of facets in education [1,2,3,4,5,6,7], including pharmacy education [8,9,10]. An important task of educators is to create clear assessment questions that reflect the learning outcomes and measure a specific knowledge level or competency [11,12,13,14]. Writing assessment questions is a thoughtful process that consumes instructors’ time [11,14,15,16,17,18,19,20]. Improving the quality of questions, updating questions to reflect changing curricular content, and protecting the integrity of examination questions are driving forces for the need to continuously reevaluate and generate new assessment questions [17,18,21].

It has been suggested that ChatGPT, a common AI chatbot, can be used to write assessment questions [2,10,12,22,23,24,25] to streamline this process and save time [15,17,19,26,27]. Information about the characteristics of ChatGPT-generated questions is needed to help educators determine whether and to what extent they wish to evaluate and use this approach. Recent reports about the use of ChatGPT to generate assessment questions generally focused on the following aspects of usability (defined as the quality and extent that enable users to achieve specific goals): Accuracy, relevance, clarity, and cognitive level, as determined by subject-matter experts [11,12,19,21,27,28,29,30], and the outcomes of administering such questions to students in assessments [1,14,16,17,19,21,24,31,32,33]. These outcomes can be assessed through psychometric analysis [1,14,19,24,31,32], which, in the context of such questions, includes item analysis metrics such as the difficulty index (the proportion of students who answer the item correctly) and the discrimination index [34]. Moreover, information on the usability of ChatGPT-generated questions has included reports on student perceptions of their quality [14,20], and the students’ ability to distinguish between ChatGPT- and instructor-generated questions [16]. Such an ability would suggest that the former need to be reevaluated, for example, in their wording, terminology, or reflection of the lecture outcomes. Some studies reported on multiple usability aspects [14,19,21].

The seminal work that described the use of ChatGPT to write assessment questions in pharmacy education was conducted by Edwards and Erstad. The study included the evaluation of ChatGPT-generated questions by subject-matter experts for clarity, formatting, relevance, and difficulty, with or without minor modifications [12]. More recently, Shultz and colleagues uploaded chapters from the DiPiro pharmacotherapy textbook to ChatGPT, aiming to generate valid case-based questions. A 38-question examination with no stakes was administered to fourth-year student pharmacists. The study encompassed a psychometric analysis of ChatGPT-generated questions, including point-biserial correlations and the difficulty index. Subject-matter experts concluded that ChatGPT could generate adequate and relevant questions, particularly after refinement [32].

ChatGPT-generated questions have also been studied in medical education [1,11,15,16,17,19,20,28,29,30,33]. Laupichler and colleagues compared student performance on ChatGPT- versus instructor-generated questions after the administration of 21 ChatGPT-generated questions in a formative assessment. The average percentage of students who selected the correct answers was 69% and 62% for the ChatGPT- and the instructor-generated questions, respectively [16]. In another study, Law and colleagues compared student performance on a mock examination (i.e., a formative assessment) comprising 100 ChatGPT-generated questions to that on a high-stakes examination comprising 100 instructor-generated questions. The difficulty index for the ChatGPT-generated questions was significantly higher, indicating that those questions were easier for students to answer. The ChatGPT-generated questions required much less time to generate. Law and colleagues also studied the levels of Bloom’s taxonomy in ChatGPT-generated questions. They reported that without specific instructions, ChatGPT defaulted to generating questions with lower Bloom’s taxonomy levels (Remember and Understand) more frequently [19].

Those studies and others show that substantial progress has been made in characterizing ChatGPT’s usability for generating assessment questions [1,11,14,15,17,20,21,24,27,28,29,30,31,33]. Nevertheless, additional research is needed on this issue. Specifically, when ChatGPT-generated questions were administered to students, the literature overwhelmingly focused on using those questions in formative assessments [1,16,17,19,21,24,31,32]. The only study we located in the literature on the use of ChatGPT-generated questions in summative assessments was by Kiyak and colleagues, who evaluated two ChatGPT-generated questions embedded in a summative assessment of a pharmacotherapy clerkship in a school of medicine, and reported on their difficulty index and point biserial [33]. Ahmed and colleagues noted that AI could be used in the future to write summative assessment questions [35].

Information about the use of ChatGPT-generated questions in summative assessments is important because it may provide examples and relevant information on better practices and student academic performance. It can also serve as proof-of-concept for instructors who consider the use of ChatGPT-generated questions in summative assessments. Moreover, we found only limited information in the medical education literature and no reports in the pharmacy education literature on the Bloom’s taxonomy levels of ChatGPT-generated questions [19,29] or on students’ ability to distinguish between ChatGPT- and instructor-generated questions [16]. Furthermore, we were unable to locate any literature on student perceptions of whether instructors should use ChatGPT to write assessment questions. Such information can be useful for instructors as they consider whether to use ChatGPT to write assessment questions and, if so, to what extent.

To reduce those gaps in the literature, this study’s objectives are to evaluate (1) student academic performance on ChatGPT-generated questions on summative assessments; (2) student ability to distinguish between ChatGPT- and instructor-generated questions, and their perceptions regarding the use of ChatGPT to generate assessment questions; and (3) Bloom’s taxonomy levels and the corresponding student performance on ChatGPT-generated questions.

## 2. Methods

### 2.1. The Educational Setting

Pharmaceutics is a pharmaceutical sciences discipline that is defined as “the science of preparing, using, or dispensing medicines” [36,37]. Pharmacy graduates are expected to have foundational knowledge in pharmaceutics, as outlined in the Curricular Outcomes and Entrustable Professional Activities [38]. Hence, pharmaceutics courses are part of Doctor of Pharmacy curricula [39,40]. The Pharmaceutics I and Pharmaceutics II courses in South College School of Pharmacy [36], an accelerated, 3-year program, are directed and taught by one of the authors (E.A.K.) during the second half of the first professional year. Pharmaceutics I is a 3-credit-hour course on a quarter system that covers fundamentals of physical pharmacy, biopharmaceutics, oral solid dosage forms (e.g., tablets and capsules), and routes of drug administration. Pharmaceutics II is a 4-credit-hour course that focuses on various dosage forms and delivery systems, including oral liquid dosage forms, drug delivery to the skin, parenterals, and ophthalmic, rectal, vaginal, pulmonary, and nasal dosage forms. The combined pharmaceutics courses encompass 70 contact hours. The course grades are based on examinations, quizzes, and assignments. Examinations are administered using ExamSoft as composite examinations that include questions from all didactic courses taught during the quarter [41]. The Pharmaceutics I course includes 5 examinations during the quarter and a final examination, whereas the Pharmaceutics II course includes 4 examinations during the quarter and a final examination.

### 2.2. Question Generation and Administration

ChatGPT-3.5 was used from December 2023 to May 2024 to generate assessment questions for inclusion in all 11 examinations for the two pharmaceutics courses. Most of the questions administered during the 2024 iterations of the courses were also administered during the 2025 iterations. The courses in 2025 included two additional topics. Five questions for these two topics were generated using ChatGPT-4o from September 2024 to March 2025. The versions of ChatGPT used were the most recent freely available versions of the platform. The prompt used for all question generation was “Please write twenty multiple-choice assessment questions with five answer choices and provide the correct answer about” followed by a lecture objective obtained from the lecture notes. Assessment questions were carefully selected from the ChatGPT output using the following criteria: Alignment with the lecture objectives, scientific accuracy, clarity, terminology similar to that used in class, a format consistent with the South College School of Pharmacy assessment policy (e.g., no “All of the above” answer choice), a single correct answer, no unintended clues regarding the answer, and an expected difficulty level (i.e., student success rate) appropriate for the course. Most of the ChatGPT-generated questions (~75%) were administered to the students without modifications [1,21,31]. For modified ChatGPT-generated questions, the change was usually limited to one word. Instructor-generated questions were selected from question banks and were expected to meet the above-mentioned criteria. The percentage of ChatGPT-generated questions was balanced across all examinations, with an average (±SD) of 38% ± 8% per examination. Academic performance on ChatGPT- and instructor-generated questions was obtained using ExamSoft tagging. Student feedback, question challenges, student success rates, and instructor re-review of examination questions led to the following changes to examination grading: For four ChatGPT-generated questions, two different answer choices were accepted as correct; no instructor-generated questions required this adjustment. Nine ChatGPT-generated and four instructor-generated questions were excluded from the examinations. Those questions did not contribute to students’ grades and were excluded from this study’s analysis. Data analysis was performed after the completion of the Pharmaceutics II course to prevent unintentional bias during question generation or selection. The use of AI in this study complied with South College’s AI policy.

### 2.3. Survey Design and Administration

A survey instrument was designed to collect demographic information and student perceptions of instructors using ChatGPT to write assessment questions, as well as to estimate students’ ability to distinguish between ChatGPT- and instructor-generated questions. For the latter, 20 examination questions from eight examinations administered during the two courses were randomly selected. Ten of the questions were ChatGPT-generated, while 10 were instructor-generated. For each of the 20 questions, the survey asked students to identify whether it was generated by the instructor or by AI (i.e., ChatGPT). Feedback on the survey was obtained from four pharmacy faculty members. After modification, the survey was pre-tested on seven student pharmacists randomly selected from the second professional year who did not participate in the study [42]. The survey without the examination questions is available in Supplementary Material S1. The South College Institutional Review Board certified the protocol as exempt. On-ground students enrolled in the Pharmaceutics II course in 2024 and 2025 were offered the opportunity to volunteer for the research project, and the survey was administered to the volunteers near the completion of the Pharmaceutics II course. In 2025, the first cohort of student pharmacists enrolled in the online pathway of South College School of Pharmacy completed the pharmaceutics courses. To avoid potential confounding introduced by differences in course delivery between the on-ground and online pathways, the online cohort was excluded from the study. The survey was administered using ExamSoft.

### 2.4. Bloom’s Taxonomy Levels

The assessment policy in the South College School of Pharmacy requires instructors to tag all examination questions in ExamSoft with Bloom’s taxonomy levels. The following modified Bloom’s taxonomy is used: Level I—Knowledge and Comprehension, Level II—Application and Analysis, and Level III—Evaluation and Synthesis [43]. The Course Director, who is a subject-matter expert in the teaching of pharmaceutics, assigned Bloom’s taxonomy levels to the assessment questions in the study.

Subsequently, CompetencyGenie™, a free Chrome extension that is an AI-powered tool developed in partnership between ExamSoft and Enflux, was used to assign Bloom’s taxonomy levels to each assessment question in the study [8,44]. CompetencyGenie™ works in conjunction with ExamSoft, and any ExamSoft user can use it. It specializes in categorizing assessment questions across various healthcare professions, including pharmacy. CompetencyGenie™ uses the revised Bloom’s taxonomy: Remember, Understand, Apply, Analyze, Evaluate, and Create [45]. In the revised taxonomy, Remember, Understand, Apply, and Analyze reflect the Knowledge, Comprehension, Application, and Analysis levels, respectively, of the original taxonomy [46]. For each question, CompetencyGenie™ also provides a brief rationale to explain its assignment to a specific Bloom’s taxonomy level.

As part of the Bloom’s taxonomy analysis, each of the examination questions was tagged according to categories that reflect the following variables: A revised Bloom’s taxonomy level of Remember, Understand, Apply, or Analyze (CompetencyGenie™ did not assign levels of Evaluate or Create to any of the assessment questions); student cohort—2024 or 2025; course—Pharmaceutics I or Pharmaceutics II; and question source—ChatGPT- or instructor-generated question. This categorization system enabled the generation of ExamSoft reports that included the number of questions and the average student score for each category.

### 2.5. Statistical Analysis

A paired *t*-test was performed to identify the differences between student performance on (a) ChatGPT- versus instructor-generated questions administered in the two courses and in the combined courses within the same academic year; and (b) questions from the same source (i.e., ChatGPT or instructor) administered in the Pharmaceutics I course versus the Pharmaceutics II course within the same academic year.

Two one-sided tests for equivalence [47] with margins of 60% and 40% around the 50% chance level were performed to assess students’ ability to distinguish between ChatGPT- and instructor-generated questions. The 60% margin was selected because various undergraduate courses have a minimum passing grade of 60%. The 40% margin was selected to achieve symmetry around a 50% chance. In this procedure, two one-tailed, one-sample *t*-tests were performed to compare the student success rate in identifying the source of the 20 examination questions on the survey to the margins. The null hypotheses were that students’ success rate would be ≥60% or ≤40%.

Fisher’s Exact Test was conducted to (a) assess the association between the student cohort and their response to a question about whether pharmacy instructors should use AI applications such as ChatGPT to construct assessment questions; and (b) assess the association between the question source (ChatGPT- and instructor-generated questions) and the revised Bloom’s taxonomy level assigned by CompetencyGenie™, within the same course taught in the same academic year.

A paired *t*-test was also used to identify differences in student scores on questions assigned the Remember versus Understand levels within the same course, question source, and academic year. The Holm-Šidák correction was used to account for multiple comparisons. Statistical significance was set at *p* < 0.05, and data were presented as the mean and standard deviation (SD), as appropriate. Statistical analysis was conducted using GraphPad Prism version 11.1.0 (224).

## 3. Results

### 3.1. Student Demographics

The response rates and demographic information for the two student cohorts who participated in the study are presented in Table 1. Information about the combined classes shows that the most frequent age group was 18–24 years. Most students self-identified as female and indicated that they hold a bachelor’s degree.

### 3.2. Student Academic Performance

The students’ average academic performance is presented in Table 2. Additional statistical data related to Table 2, Table 3 and Table 4, Figure 1, and students’ ability to distinguish between ChatGPT- and instructor-generated questions are provided in Supplementary Material S2. The students were graded on approximately 100 ChatGPT-generated questions for the combined courses. In both 2024 and 2025, in the Pharmaceutics I course, the average percent correct was higher on ChatGPT-generated questions compared to those created by the instructor, whereas in the Pharmaceutics II course, the percent correct was lower on ChatGPT-generated questions. Student performance on instructor-generated questions was higher in the Pharmaceutics II course compared to the Pharmaceutics I course. In 2024, student performance on ChatGPT-generated questions was higher in the Pharmaceutics I course compared to the Pharmaceutics II course. For the combined courses, the percent correct was significantly lower for ChatGPT-generated questions compared to instructor-generated questions.

### 3.3. Student Perceptions

The survey asked students to select True or False in response to the following statement: “I noticed throughout the examinations that some of the questions may have been written by an artificial intelligence (AI) application such as ChatGPT.” In 2024, 2025, and for the combined classes, 77%, 71%, and 74% of students, respectively, selected the False option.

Student perceptions regarding the statement, “Pharmacy instructors can/should use artificial intelligence (AI) applications such as ChatGPT to construct assessment questions (i.e., examination, quiz) for which part of their assessment questions?” are presented in Figure 1. Most students did not support instructors using ChatGPT to generate most (≥50%) or all of their assessment questions; specifically, 95% and 79% of students from the 2024 and 2025 cohorts, respectively, agreed that instructors should limit the use of ChatGPT to generate some (<50%) or none of the assessment questions. There was a significant association between the cohort and the response to this question (*p* < 0.05). In the 2025 cohort, students were more accepting of instructors using ChatGPT to generate assessment questions.

Students were asked to identify, for 20 survey questions, whether they were ChatGPT- or instructor-generated. For 2024, 2025, and the combined classes, the average (±SD) percent correct was 55% ± 11%, 52% ± 12%, and 53% ± 11%, respectively. Both null hypotheses—that the mean student success rate was ≥60% or ≤40%—were rejected (*p* < 0.05). This indicated that, on average, students’ ability to distinguish between ChatGPT- and instructor-generated questions was equivalent to random chance.

### 3.4. Bloom’s Taxonomy Levels

The Course Director assigned a Level I—Knowledge and Comprehension on the modified Bloom’s taxonomy to all assessment questions in the study. A summary of the assignment of the revised Bloom’s taxonomy levels by CompetencyGenie™ is seen in Table 3. At least 96% of the ChatGPT-generated questions for each course iteration were assigned levels of Remember and Understand on the revised Bloom’s taxonomy [45,46]. Those levels correspond well with the Bloom’s taxonomy assignment of the Course Director. The statistical analysis showed a significant association between the question source (i.e., ChatGPT- or instructor-generated) and the revised Bloom’s taxonomy level in the Pharmaceutics I course taught in 2024 (*p* < 0.05). CompetencyGenie™ assigned to 14% of the instructor-generated questions in this course iteration a Bloom’s taxonomy level of Analyze. The Course Director disagrees with this part of CompetencyGenie™’s assignment of Bloom’s taxonomy levels.

A summary of student academic performance on questions grouped by revised Bloom’s taxonomy levels is shown in Table 4. There were no significant differences between the student scores on questions assigned the Remember level versus the Understand level for ChatGPT-generated questions administered within the same course and academic year. There was a significant difference between the student performance on questions assigned the Remember versus the Understand levels for instructor-generated questions administered in the Pharmaceutics II course for the 2024 cohort (*p* < 0.05).

## 4. Discussion

### 4.1. The Context of the Study

This study aimed to contribute to the characterization of the usability of ChatGPT-generated assessment questions by evaluating aspects with very limited or no prior reporting in the literature. As part of the analysis of student performance on summative assessments, we report student performance on approximately 100 ChatGPT-generated questions administered across 11 summative assessments for each of the two student cohorts. Second, we report on students’ ability to distinguish between ChatGPT- and instructor-generated questions on questions that students encountered earlier as part of their summative assessments. Moreover, we describe student perceptions regarding the use of ChatGPT-generated questions. Furthermore, to our knowledge, this is the first study regarding student performance on ChatGPT-generated questions assigned with Bloom’s taxonomy levels.

### 4.2. Student Academic Performance

The use of ChatGPT to generate assessment questions was part of an overhaul of the two pharmaceutics courses’ question banks that also included changes to instructor-generated questions. Some of those questions were used in previous years in similar versions. However, as a group of questions, both the ChatGPT- and instructor-generated questions were administered for the first time in 2024.

The differences in student performance on instructor-generated questions for the two courses suggest that the difficulty levels of those questions varied between the two courses. Therefore, differences in student performance on ChatGPT- and instructor-generated questions across the two courses could not be attributed solely to differences in the difficulty level of ChatGPT-generated questions.

There are several possible reasons for better student performance on ChatGPT-generated questions in the Pharmaceutics I course compared to the Pharmaceutics II course, as seen for the 2024 cohort: The nature of the material taught in the two courses, and the learning outcomes, differed in that the Pharmaceutics I course contained more theoretical and abstract topics related to physical pharmacy compared to the Pharmaceutics II course, where the primary focus is on the various dosage forms and delivery systems [36]. It is possible that the questions ChatGPT generated for topics of different natures varied in difficulty. Moreover, the question-generation process and subsequent selection were continuous and spanned several months. During that time, changes to the ChatGPT platform may have led to questions of varying difficulty levels.

At the course level, students’ overall examination performance was rather consistent. Differences in student performance across the various question sources or for questions with the same source in different courses were revealed only during data analysis, after completion of the Pharmaceutics II course. While some statistical significance was detected in the comparisons between ChatGPT- and instructor-generated questions, those differences were mostly small in magnitude. In particular, when data from both courses were combined, differences in performance between question sources were only 1–2%. Such differences, even if statistically significant, do not necessarily indicate meaningful educational differences [48]. Overall, student performance on the ChatGPT-generated questions supports the potential usefulness of this approach. It is noteworthy that reports in the literature on student performance on formative assessments have shown variability in the outcomes of their comparisons of the difficulty index of ChatGPT- and instructor-generated questions: In different studies, ChatGPT-generated questions tended to have higher [19,24], comparable [16,17], or lower difficulty indices [21,31].

### 4.3. Student Perceptions

Student perceptions of instructors using ChatGPT to generate assessment questions were more receptive in 2025 than in 2024 (Figure 1). This difference may be related to the increasing familiarity and acceptance of AI among students [4,49].

Most students reported that during the courses, they did not notice that an AI platform such as ChatGPT may have generated some of the questions. Moreover, the results indicated that when students were presented with assessment questions from their previous examinations, their ability to distinguish between ChatGPT- and instructor-generated questions was, on average, equivalent to random chance. This information further supports the potential usefulness of ChatGPT to generate assessment questions.

### 4.4. Bloom’s Taxonomy Levels

The Course Director assigned all ChatGPT-generated questions in the study to Level I—Knowledge and Comprehension on the modified Bloom’s taxonomy. CompetencyGenie™ was used to obtain further, unbiased insights regarding this issue. Similar to the Course Director, CompetencyGenie™ assigned revised Bloom’s taxonomy levels of Remember and Understand to most assessment questions. Student performance on questions assigned Remember versus Understand levels from the same question source, and administered within the same course and academic year, was usually similar. There are reports, unrelated to ChatGPT-generated questions, which indicate that student performance on the Remember and Understand levels was similar [50,51,52]. It was also noted that the hierarchy level on Bloom’s taxonomy is not directly analogous to the difficulty level of the questions [50,51,53].

While the questions selected and administered to the students were evidently usable, the study did not aim to obtain assessment questions for specific Bloom’s taxonomy levels. Therefore, Bloom’s taxonomy levels or its specific terminology (e.g., comprehension, knowledge) were not mentioned in the prompt that was used. There are recommendations in the literature to improve ChatGPT’s assessment question generation by the inclusion of verbiage regarding desired Bloom’s taxonomy levels in the prompt [30,54]. Nevertheless, it is noteworthy that various other reports about the use of ChatGPT to generate assessment questions also did not describe the inclusion of Bloom’s taxonomy in their prompts [1,11,12,14,15,16,17,26,27,31,32,33,55].

In the study, prompting ChatGPT without specific verbiage about Bloom’s taxonomy levels yielded assessment questions that mostly aligned with the Remember and Understand levels. As indicated earlier, a similar phenomenon was previously reported in the literature regarding ChatGPT-generated questions generated for medical education [19]. Nevertheless, the literature also suggests that prompt modifications can lead ChatGPT to generate questions with various Bloom’s taxonomy levels [29,30].

### 4.5. Study Limitations

This study has several limitations. First, we evaluated ChatGPT’s ability to generate examination questions in pharmaceutics. Therefore, the findings may not apply to other pharmacy or healthcare-related disciplines. Second, this study was conducted at a single institution on a relatively small number of students, thus limiting the generalization of its conclusions to other institutions and student groups [1,31,33,36]. Furthermore, most of the ChatGPT assessment questions in this study were generated using ChatGPT-3.5, while later versions of ChatGPT have enhanced capabilities [19,54,56,57,58]. The question generation did not include the uploading of lecture notes, although it is possible that doing so would yield better results with ChatGPT [17,27]. Repeated prompting of ChatGPT yields different outputs [58]. Therefore, it would be impossible to replicate the same set of assessment questions obtained from ChatGPT in the study. CompetencyGenie™ and a subject-matter expert assigned similar Bloom’s taxonomy levels to the assessment questions in the study. However, we were unable to locate reports in the literature on the accuracy of CompetencyGenie™ in assigning Bloom’s taxonomy levels to assessment questions, for example, by comparing its assignments with those of a group of subject-matter experts.

Another study limitation is that a detailed psychometric analysis was not reported. Most psychometric metrics, including discrimination index, point biserial, and Kuder–Richardson formula 20 (KR-20), are calculated taking into account the student performance across a set of questions [59]. In ExamSoft, even after tagging questions to a specific course (e.g., a pharmaceutics course), psychometric analysis of most metrics considers student performance across all examination questions in the composite examination, rather than only those tagged to that course. Therefore, after the administration of composite examinations, most of the output from the ExamSoft psychometric analysis is not publishable. Despite the study limitations, it is a step forward in characterizing the usability of ChatGPT to generate assessment questions.

## 5. Conclusions

ChatGPT can generate questions that, after content-expert review and selection, are usable in summative assessments of pharmaceutics courses. Future directions in using AI to generate assessment questions include evaluating various AI platforms beyond ChatGPT for this practice; engineering better prompts; assessing this practice in various pharmacy and healthcare-related courses; and obtaining more information regarding item analysis of such questions.

## Figures and Tables

**Figure 1:**
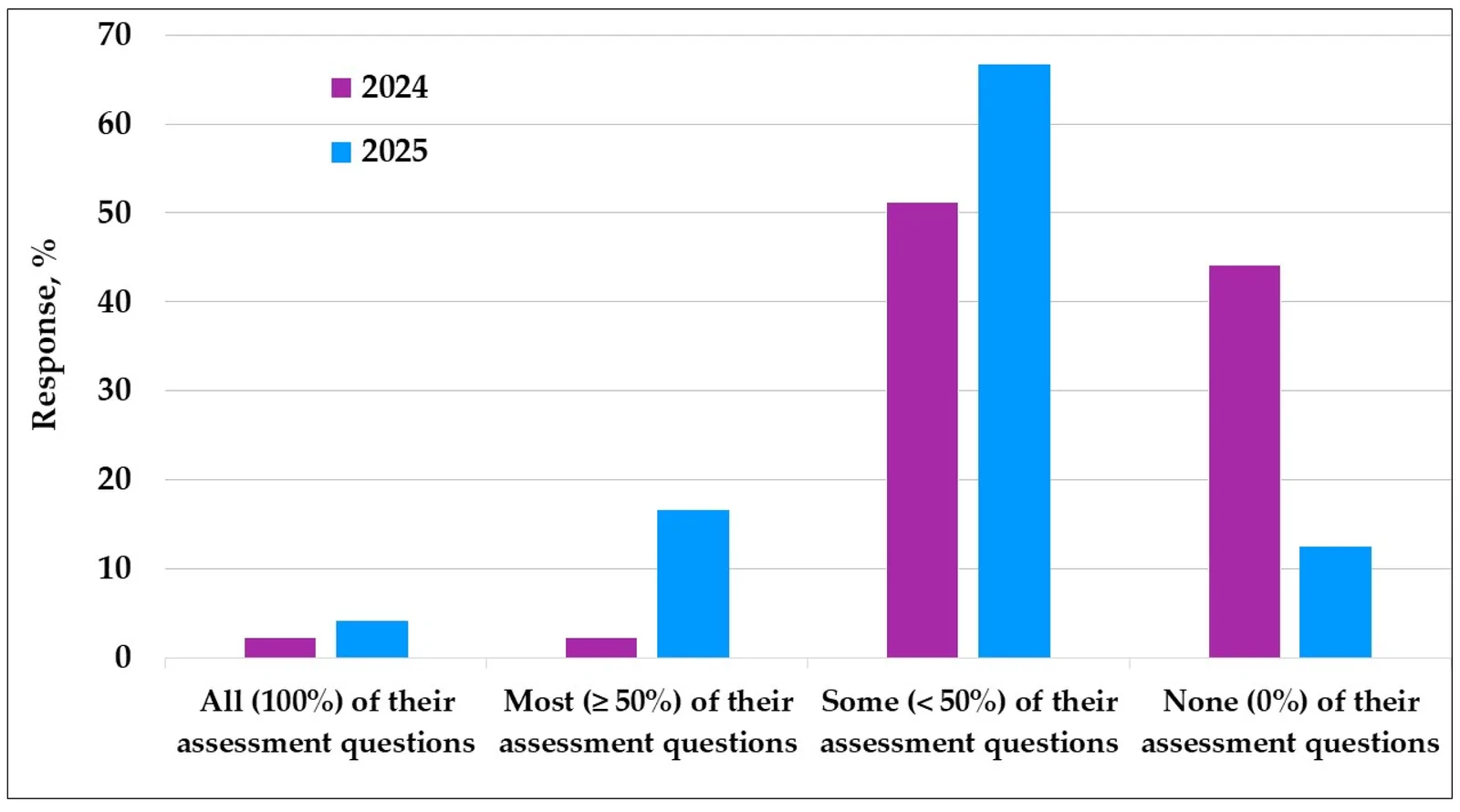
Student response to the following survey question: Pharmacy instructors can/should use artificial intelligence (AI) applications such as ChatGPT to construct assessment questions (i.e., exam, quiz) for which part of their assessment questions? There was a significant association between the cohort and the response to this question. *p* < 0.05, 2024 cohort (n = 43), 2025 cohort (n = 48).

**Table 1:** Student demographics (2024, n = 43; 2025, n = 48; combined = 91).

		2024 Cohort, %	2025 Cohort, %	Combined Classes, %
Response rate		90	96	93
Age, years	18–24	49	38	43
	25–30	37	40	38
	≥31	14	23	19
	Prefer not to answer	0	0	0
Gender	Female	77	69	73
	Male	23	31	27
	Non-binary	0	0	0
	Transgender	0	0	0
	Prefer not to answer	0	0	0
Highest earned academic degree	No degree	14	6	9
	Associate	28	23	25
	Bachelor’s	49	65	57
	Master’s	9	6	7
	PhD	0	0	0

**Table 2:** Student academic performance.

		2024 Cohort, n = 41 ^a^		2025 Cohort, n = 48	
Course	Question Source	Number of Questions	Percent Correct, Mean ± SD	Number of Questions	Percent Correct, Mean ± SD
Pharmaceutics I	ChatGPT	42	78 ± 8 ^b,c^	47	76 ± 11 ^b^
	Instructor	74	75 ± 11 ^d^	77	73 ± 11 ^d^
	ChatGPT and instructor	116	76 ± 10	124	74 ± 11
Pharmaceutics II	ChatGPT	55	73 ± 10 ^b^	56	74 ± 13 ^b^
	Instructor	82	79 ± 11	84	79 ± 11
	ChatGPT and instructor	137	76 ± 10	140	77 ± 11
Combined courses	ChatGPT	97	75 ± 9 ^b^	103	75 ± 11
	Instructor	156	77 ± 11	161	76 ± 11
	ChatGPT and the instructor	253	76 ± 10	264	76 ± 10

Abbreviation: SD, standard deviation. ^a^ Two students who were enrolled in the Pharmaceutics II course in 2024 and volunteered for the study took the Pharmaceutics I course in 2023. Since their academic performance on ChatGPT-generated questions was incomplete, their scores were not included in the analysis of the 2024 data. ^b^ *p* < 0.05, ChatGPT-generated questions compared to instructor-generated questions from the same course and academic year. ^c^ *p* < 0.05, ChatGPT-generated questions for the Pharmaceutics I course compared to the Pharmaceutics II course for the same academic year. ^d^ *p* < 0.05, instructor-generated questions for the Pharmaceutics I course compared to the Pharmaceutics II course for the same academic year.

**Table 3:** Revised Bloom’s taxonomy levels of ChatGPT- and instructor-generated questions as assigned by CompetencyGenie™ ^a^.

Course	Question Source	Bloom’s Level ^b^	2024, Number of Questions, %	2025, Number of Questions, %
Pharmaceutics I	ChatGPT ^c^	Remember	25, 60%	27, 57%
		Understand	17, 40%	18, 38%
		Apply	0, 0%	1, 2%
		Analyze	0, 0%	1, 2%
	Instructor	Remember	24, 32%	26, 34%
		Understand	40, 54%	46, 60%
		Apply	0, 0%	1, 1%
		Analyze	10, 14%	4, 5%
Pharmaceutics II	ChatGPT	Remember	34, 62%	32, 57%
		Understand	19, 35%	22, 39%
		Apply	0, 0%	0, 0%
		Analyze	2, 4%	2, 4%
	Instructor	Remember	40, 49%	43, 51%
		Understand	33, 40%	31, 37%
		Apply	4, 5%	4, 5%
		Analyze	5, 6%	6, 7%

^a^ ComptencyGenie™ is a free Chrome extension powered by artificial intelligence that works in conjunction with ExamSoft to categorize assessment questions of healthcare professions [44]. ^b^ CompetencyGenie™ assigned to the assessment questions only Bloom’s taxonomy levels of Remember, Understand, Apply, and Analyze. No questions were assigned the Evaluate or Create levels. ^c^ *p* < 0.05, the question source (i.e., ChatGPT- or instructor-generated questions) was significantly associated with the revised Bloom’s taxonomy level for the Pharmaceutics I course taught in 2024.

**Table 4:** Student academic performance on questions according to their revised Bloom’s taxonomy levels obtained from CompetencyGenie™ ^a^.

Course	Question Source	Bloom’s Level ^b^	2024, Percent Correct (Mean ± SD)	2025, Percent Correct (Mean ± SD)
Pharmaceutics I	ChatGPT ^c^	Remember	76 ± 11	75 ± 13
		Understand	81 ± 10	78 ± 12
		Apply	N/A ^c^	N/A ^c^
		Analyze	N/A ^c^	N/A ^c^
	Instructor	Remember	72 ± 13	74 ± 13
		Understand	77 ± 13	72 ± 13
		Apply	N/A ^c^	N/A ^c^
		Analyze	77 ± 13	68 ± 21
Pharmaceutics II	ChatGPT	Remember	73 ± 10	73 ± 12
		Understand	73 ± 13	75 ± 15
		Apply	N/A ^c^	N/A ^c^
		Analyze	N/A ^c^	N/A ^c^
	Instructor	Remember	81 ± 11 ^d^	78 ± 12
		Understand	76 ± 14	79 ±12
		Apply	87 ± 17	79 ± 21
		Analyze	75 ± 21	75 ± 19

Abbreviations: N/A, not available; SD, standard deviation. ^a^ CompetencyGenie™ is a free Chrome extension powered by artificial intelligence that works in conjunction with ExamSoft to categorize assessment questions of healthcare professions [44]. ^b^ CompetencyGenie™ assigned to the assessment questions only revised Bloom’s taxonomy levels of Remember, Understand, Apply, and Analyze. No questions were assigned the Evaluate or Create levels. ^c^ Percent correct on categories that included <3 assessment questions are not presented. ^d^ *p* < 0.05, percent correct on questions assigned the Remember level were different from those assigned the Understand level for the same course, question source, and academic year.

## Data Availability

The data presented in this study are available on request from the corresponding author. The data are not publicly available due to participant privacy and confidentiality restrictions.

## Supplementary Materials

The following supporting information can be downloaded at: https://www.mdpi.com/article/10.3390/pharmacy14070142/s1, Supplementary Material S1: Survey instrument; Supplementary Material S2: Statistical data.

## Abbreviations

The following abbreviations are used in this manuscript:

AI	Artificial intelligence
SD	Standard deviation
