# Supplementary material for: The Use of ChatGPT to Write Assessment Questions
Source: Pharmacy (Basel). 2026 Sep 24;14(7):142. doi: 10.3390/pharmacy14070142 (PMC13708386; doi:10.3390/pharmacy14070142)
Supplement: Supplementary file 1 [file pharmacy-14-00142-s001.zip › pharmacy-4488387-Supplementary Material S1 survey instrument.pdf]

Supplementary Material S1: Survey instrument

A survey about the use of ChatGPT to write assessment questions

1. What is your age?

A. 18-24

B. 25-30

C. ≥ 31

D. Prefer not to answer

2. What is your gender?

A. Female

B. Male

C. Non-binary

D. Transgender

E. Prefer not to answer

3. What is the highest academic degree you earned? (Not including your current enrollment in a Doctor of Pharmacy degree)

A. No degree

B. Associate

C. Bachelor

D. Master

E. PhD

4. Pharmacy instructors can/should use artificial intelligence (AI) applications such as ChatGPT to construct assessment questions (i.e., exam, quiz) for which part of their assessment questions?

A. All (100%) of their assessment questions.

B. Most (≥ 50%) of their assessment questions.

C. Some (< 50%) of their assessment questions.

D. None (0%) of their assessment questions.

5. I noticed throughout the exams that some of the questions may have been written by an artificial intelligence (AI) application such as ChatGPT.

A. True

B. False

The following 20 questions are related to questions that appeared on various exams of the Pharmaceutics I and Pharmaceutics II courses; please answer to the best of your ability.

---

6. The following question

> Which of the following is an example of an electrolyte?
> A. Sucrose
> B. Glycerin
> C. Tadalafil
> D. Ethanol
> E. Hydrochloric Acid

was written by:

A. The instructor (i.e., Dr. Klausner)

B. Artificial Intelligence (i.e., ChatGPT)

[There are 19 additional questions similar in structure to question 6, but with a different exam question]
